# The associations between air pollution and hippocampal volume: The Betula Project

**DOI:** 10.1002/alz.093220

**Published:** 2025-01-09

**Authors:** Shireen Sindi, Micael Andersson, Mikael Stiernstedt, Anna Oudin, Lars Nyberg

**Affiliations:** ^1^ Karolinska Institutet, Department of Neurobiology, Care Sciences and Society, Division of Clinical Geriatrics, Center for Alzheimer Research, Stockholm Sweden; ^2^ The Ageing Epidemiology (AGE) Research Unit, School of Public Health, Imperial College London, London United Kingdom; ^3^ Umeå Center for Functional Brain Imaging, Umeå University, Umeå Sweden; ^4^ Department of Medical and Translational Biology, Umeå University, Umeå Sweden; ^5^ Division of Occupational and Environmental Medicine, Department of Laboratory Medicine, Faculty of Medicine, Lund University, Lund, Sweden., Lund Sweden; ^6^ Department of Department of Diagnostics and Intervention, Umeå University, Umeå Sweden

## Abstract

**Background:**

Long‐term exposure to ambient air pollution has recently been highlighted as a modifiable risk factor for dementia. However, the mechanisms underlying these associations still remain unclear. The goal of this study was to investigate the associations between air pollution and neuroimaging correlates in a sample of middle‐aged and older adults.

**Methods:**

This study used data from the Betula Project, which is a longitudinal study on aging, memory and dementia in Umeå, Northen Sweden. Participants were aged 50‐81 years old. For air pollution measures, data were available for local and total fine ambient particulate matter (PM_2.5_, PM_10_) and black carbon (BC) from vehicle exhaust and wood‐smoke. Participants were scanned in a 3T Discovery 750 (General Electric) MR‐scanner. All T1‐images were processed through a longitudinal pipeline in Freesurfer ver 7.11. MRI outcomes included total grey volume, cortex thickness, ventricle volume, and hippocampus volume. After removing participants due to incorrect hippocampus segmentation or missing values in air pollution or nuisance regressors 249 participants were included in the analyses. Partial correlations were performed, adjusting for age, sex and education. Additional analyses were conducted, stratifying by APOE4 status and sex.

**Results:**

The analyses showed that smaller hippocampal volume was associated with higher levels of all three air pollution measures (PM_2.5_ (r = ‐0.159, p = 0.012), PM_10_ (r = ‐0.125, p = 0.048), and BC (r = ‐0.148, p = 0.019)). In the APOE4 non‐carrier group, smaller hippocampal volume was associated with higher PM2.5 (r = ‐159, p = 0.034), whereas this association was not significant among the APOE4 carriers (r = ‐0.161, p = 0.178). The association between smaller hippocampal volume and PM2.5 was more pronounced among women (r = ‐0.168, p = 0.052) compared to men (r = ‐0.150, p = 0.112)

**Conclusion:**

This study shows that ambient air pollution is associated with smaller hippocampal volume, and this association appears to be more pronounced among APOE4 non‐carriers and women. These results shed light on the mechanism through which air pollution may increase the risk for cognitive decline and dementia, with important implications for risk reduction initiatives.

